# The effect of ice ingestion during endurance exercise

**DOI:** 10.1186/2046-7648-4-S1-A126

**Published:** 2015-09-14

**Authors:** Gaizka Mejuto, Stephanie Gilbert, Sam Chalmers, Roger Eston, David Bentley

**Affiliations:** 1 Human Performance Laboratory, Department of Sport and Physical Education, University of the Basque Country, Vitoria-Gasteiz, Spain

## Introduction

When exercising in a hot and humid environment, athletes encounter thermoregulatory strain leading to decreased central neurological drive and reduced skeletal muscle activation [1]. Reducing initial core temperature prior to exercise increases the capacity to store metabolic and environmental heat [2]. It has been also found that ice 'slurry' ingestion is an effective precooling strategy reducing rectal temperature (T_re_) [5]. The purpose of this study was to compare the effects of ice slurry ingestion before exercise in the heat with combined precooling (with ice slurry) and ingestion of ice slurry *during *exercise on endurance performance in hot conditions.

## Methods

Eight well-trained, male cyclists (age = 33.7(9.2) yrs, VO_2 _max = 58.3(5.9) participated in the study. Participants were allocated an order of trials in a randomised crossover design. Trials were (1) no cooling before or during exercise (*thermo-neutral*, TN), (2) precooling and no cooling during exercise (*precooling only*, PO), or (3) precooling and cooling during exercise (*precooling and cooling during*, PCD). The experimental trials took place in hot environmental conditions (32 °C, 50% relative humidity) in an environmental chamber. In these conditions participants exercised on a cycle ergometer at 70% VO_2 _max (SS) for 45 mins, followed by a 10 km time trial (TT), using self-selected cadence and intensity. The effects of the different cooling strategies on each physiological variable were assessed using a two way ANOVA (trial × time). For all comparisons, significance was set at p = 0.05.

## Results

There were no significant differences in performance between the conditions in the 10 km TT performance (TN: 14.90(0.99), PO: 15.2(1.14), PCD: 15.30(1.15) mins, p = 0.72) but T_re _was significantly lower in PO and PCD than TN during SS cycling (p < 0.05) and in the TT (p < 0.05).

## Discussion

These findings contrast recent studies which report ice slurry ingestion to improve endurance performance in the heat [4]. A common variation in these studies from the present study is the duration of the exercise protocol. However, in the present study, ingested ice-slurry is an effective way in reducing T_re _as other studies have previously reported [3].

## Conclusion

The ingestion of ice slurry may be a practical and effective way of cooling the body before and during exercise. This may be beneficial in reducing occurrences of heat stress. Additionally, this study showed that the cooling measures may only be beneficial to performance in longer duration exercise when an athlete is under high levels of heat stress.

References:
